# Incidental Finding of Secondary Tumoral Calcinosis Following Cardiothoracic Surgery: The Role of Multimodality Imaging Including Spectral Detector Computed Tomography

**DOI:** 10.7759/cureus.26929

**Published:** 2022-07-16

**Authors:** Mihnea-Alexandru Găman, Mohamed M Gad, Najdat Bazarbashi, Robert Gilkeson, Amit Gupta

**Affiliations:** 1 Medicine, "Carol Davila" University of Medicine and Pharmacy, Bucharest, ROU; 2 Hematology, Center of Hematology and Bone Marrow Transplantation, Fundeni Clinical Institute, Bucharest, ROU; 3 Cardiovascular Medicine, Baylor College of Medicine, Houston, USA; 4 Cardiovascular Medicine, Georgetown University School of Medicine, MedStar Washington Hospital Center, Washington, USA; 5 Radiology, University Hospitals Cleveland Medical Center, Cleveland, USA; 6 Diagnostic Radiology, Case Western Reserve University School of Medicine, Cleveland, USA

**Keywords:** spectral detector computed tomography, end-stage renal disease, renal failure, hyperparathyroidism, tumoral calcinosis

## Abstract

Tumoral calcinosis is a rare syndrome that affects mostly soft tissues. It is characterized by calcium salt deposition in the periarticular soft tissue surrounding bony structures forming slow-growing, seldom asymptomatic masses. This case report describes a 41-year-old male with end-stage renal disease on home hemodialysis, who presented with an unusual rapidly progressive mass overlying the manubrium and suprasternal notch, following recent cardiothoracic surgery, which was initially felt to be a hematoma. The case highlights the role of spectral detector computed tomography (SDCT) in reaching the correct diagnosis of tumoral calcinosis as well as demonstrating additional changes of ectopic parathyroid hyperplasia in the anterior mediastinum.

## Introduction

Tumoral calcinosis (TC) is a rare, complex, and benign entity, primarily affecting the surrounding soft tissues of adjacent joints, which was first reported by Inclan et al. in 1943 [1]. The pathophysiology of TC involves the deposition of calcium salts in soft tissues as a result of metabolic deregulation of the parathyroid gland-renal pathway. TC can be classified into primary or secondary. Primary TC is caused by familial genetic mutations, whereas, secondary TC is due to secondary and tertiary hyperparathyroidism induced by end-stage renal disease (ESRD) [2].

TC lesions are most notably well-demarcated, firm, painless, and lobulated masses that locate mostly near joints, and rarely in the neck and head. In this case report, we present a rare and unique case presentation of the entity where spectral detector computed tomography (SDCT) played a vital role in the diagnosis and thus avoiding patient morbidity.

## Case presentation

**Figure 1:**
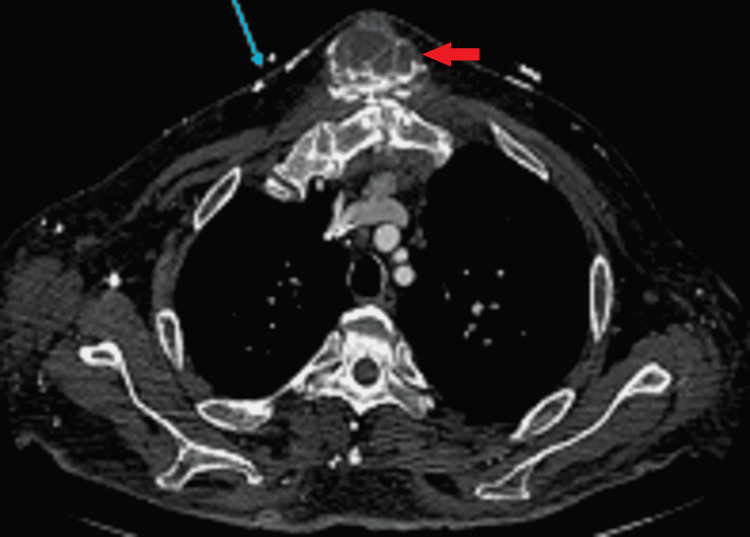
Initial axial contrast-enhanced conventional CT image demonstrating a multilobulated lesion in the soft tissues immediately superficial to sternotomy site with hyperattenuating layering material (red arrow) and reported as a hematoma with contrast material suggesting active bleed, especially given adjacent chest wall collaterals (blue arrow) from chronic right-sided central venous obstruction.

**Figure 2:**
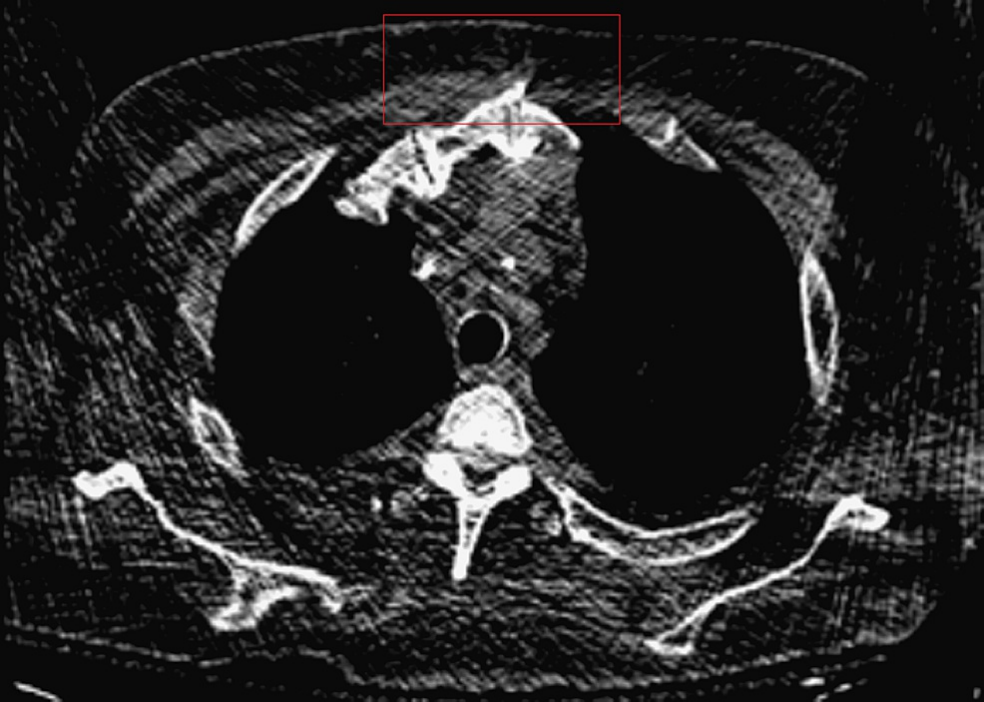
Chest CT scan from five months prior is without evidence of anterior chest wall collection (the red box represents the expected site of the superficial lesion in Figure 1).

A 41-year-old male has a past medical history of hypertension, hyperlipidemia, type 2 diabetes mellitus, ESRD on home hemodialysis five days a week since 2014, and coronary artery disease status post-three-vessel coronary artery bypass grafting as well as simultaneous mitral and tricuspid valves repair, six months before presentation. He presented with a large palpable, painless, swelling overlying manubrium and supraclavicular notch, which was firm in consistency with no clear definition of the edges and no overlying skin changes. It was non-pulsatile, non-tender, and measured approximately 15-20 cm. The sternotomy wound was completely healed. There were no signs of sternal dehiscence. Posterior-anterior and lateral chest x-rays were obtained initially and did not show the lesion despite repeated retrospective review of the x-rays; the nonspecific soft tissue swelling could not be confidently connected to the clinically palpated lesion. Due to the abnormal physical findings, a contrast-enhanced CT scan of the chest was requested for further evaluation. Initial conventional CT demonstrated a multilobulated lesion in the soft tissues immediately superficial to the sternotomy site with hyperattenuating layering material (Figure 1), which was new compared to a previous CT scan from five months ago (Figure 2) and initially reported as a hematoma with contrast material suggesting active bleeding.

**Figure 3:**
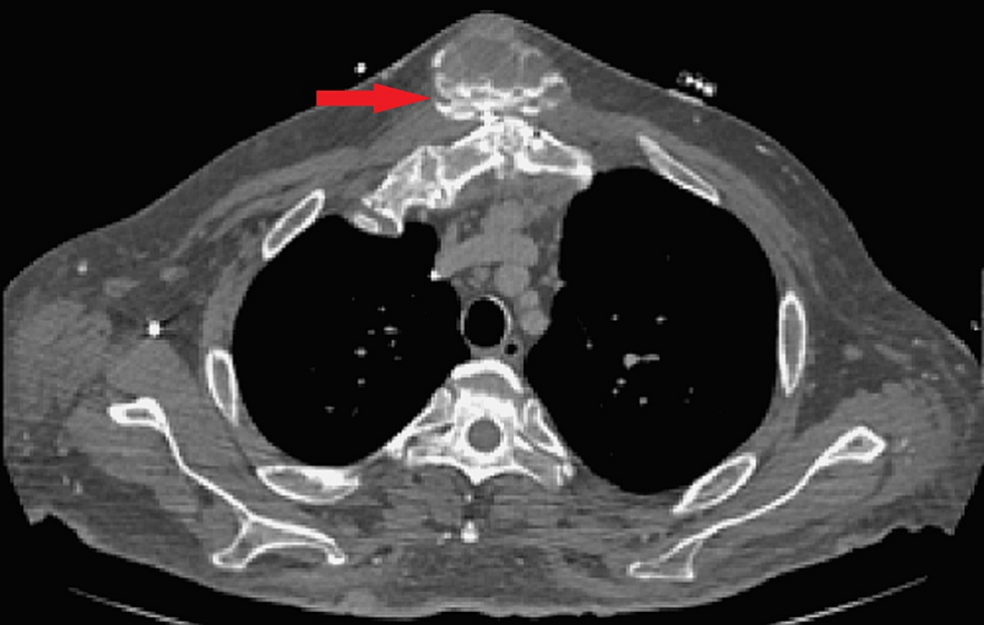
Virtual non-contrast (VNC) image from SDCT showing the persistence of layering hyperdensity (red arrow) consistent with calcification (typical “sedimentation sign”), and suggesting the diagnosis of tumoral calcinosis.

However, due to the discrepancy in the clinical and imaging findings, the images were reviewed again. As the exam was performed on SDCT, multiple reconstructions were retrospectively available for analysis. On the virtual non-contrast (VNC)/no iodine image (Figure 3) from SDCT, there was a persistence of layering hyperdensity, consistent with calcification (typical “Sedimentation sign”), and diagnosis of TC was suggested.

**Figure 4:**
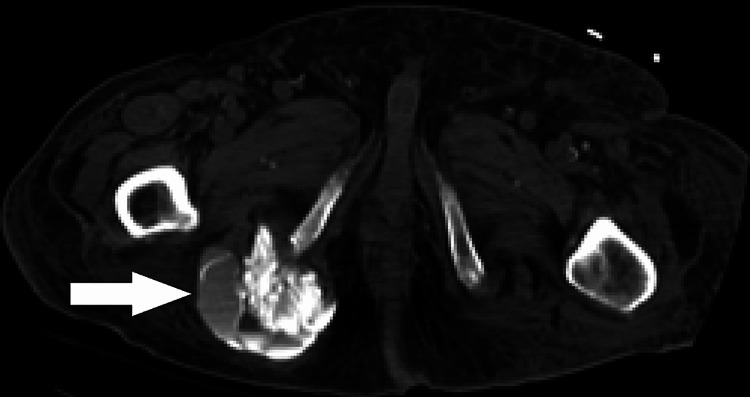
Non-contrast CT of the abdomen and pelvis one year before the current presentation depicting layering hyperdense material in the right pelvic cystic lesion with fluid calcium levels (white arrow), consistent with tumoral calcinosis.

**Figure 5:**
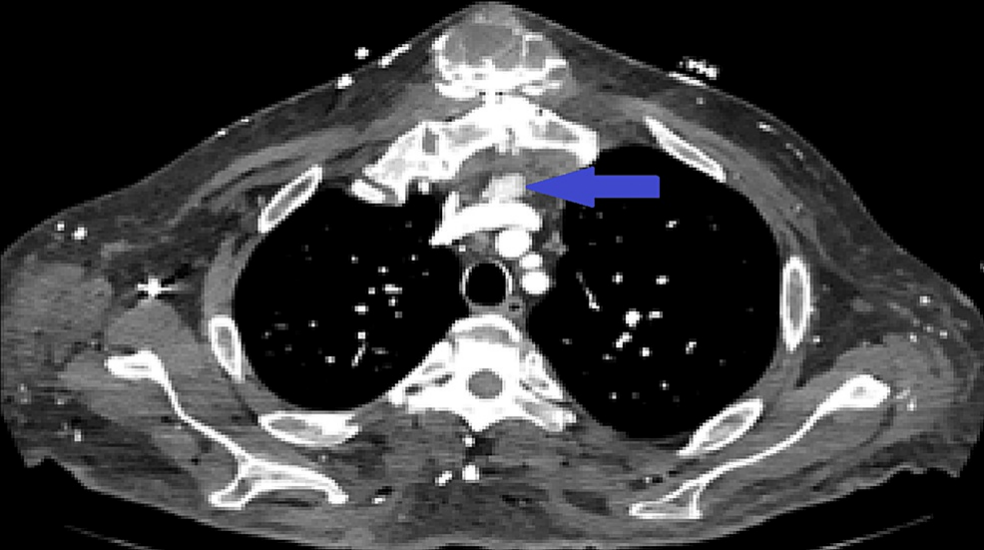
Low energy virtual monoenergetic (VMIlow) image from SDCT provides with contrast boost and better visualization of the anterior mediastinal enhancing nodule (blue arrow), which was suspicious for ectopic parathyroid hyperplasia in the setting of ESRD.

The diagnosis was also supported by the similar layering of hyperdense material in a cystic lesion with fluid calcium levels in the right pelvis on an outside non-contrast CT of the abdomen and pelvis performed one year before the current presentation (Figure 4). Additionally, a low virtual monoenergetic (VMIlow) image (Figure 5) from SDCT, provided with contrast boost and better visualization of an anterior mediastinal enhancing nodule, which was missed on the initial conventional CT image interpretation and suspicious for ectopic parathyroid hyperplasia in the setting of ESRD.

**Figure 6:**
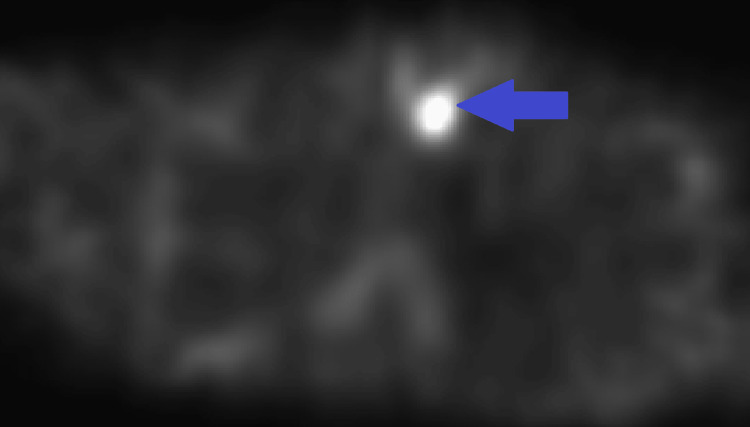
Single photon emission computed tomography (SPECT) images from Tc-99m sestamibi parathyroid nuclear medicine scan, with focal radiotracer uptake corresponding to an anterior mediastinal nodule (blue arrow) confirming the diagnosis of parathyroid hyperplasia.

**Figure 7:**
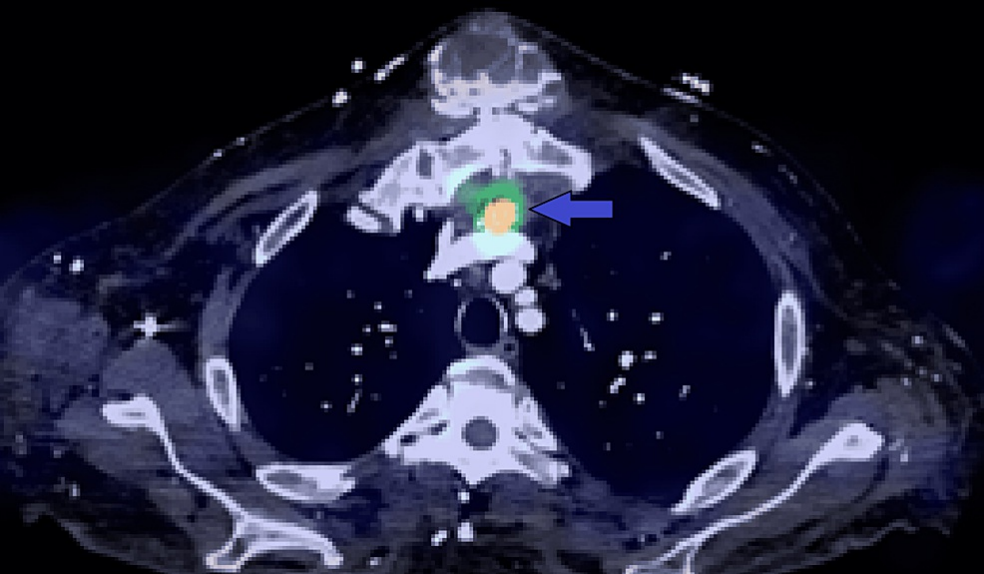
Retrospective image fusion of low VMI from SDCT and SPECT data shows the anterior mediastinal nodule and radiotracer uptake more clearly (blue arrow).

Laboratory testing and subsequent Tc-99m sestamibi parathyroid nuclear medicine scan (Figures 6, 7) confirmed the suspicion; calcium level was normal at 8.9 mg/dL (normal range, 8.6-10.6 mg/dL), phosphate level was elevated at 7.8 mg/dL (normal range, 2.5-4.9 mg/dL), and parathyroid hormone (PTH) level was significantly higher than normal at 2,300 pg/mL (normal range, 18.5-88 pg/mL). The findings are consistent with tertiary hyperparathyroidism from ESRD.

Finally, the patient underwent subtotal parathyroidectomy with the removal of enlarged left and right superior and ectopic left inferior parathyroid glands. Normal appearing right inferior parathyroid gland was left behind. The presternal mass was also removed as part of the median sternotomy for resection of the mediastinal parathyroid gland. The mass contained thick consistency pus-like fluid, and histopathologic analysis showed calcified nodules separated by fibrovascular bands consistent with a diagnosis of TC.

Post-operatively, the patient was placed on a calcium gluconate drip due to low serum calcium levels and the risk of hungry bone syndrome (which is defined as rapid, profound, and prolonged hypocalcemia following parathyroid surgery). On the fourth post-op day, the IV calcium infusion was stopped, and he was transitioned to oral calcium supplements. On discharge, the patient's serum ionized calcium was slightly low but stable on a regimen of 5 g calcium carbonate four times a day, along with cinacalcet and calcitriol.

## Discussion

Our current case highlights the role of spectral CT in diagnosing and managing a rare entity, TC, in a patient with a history of ESRD and open-heart surgery, as well as detection of additional anterior mediastinal abnormality, compatible with parathyroid hyperplasia secondary to renal dysfunction. SDCT data informed the further management of this case, as establishing the diagnosis was crucial to managing the condition with parathyroidectomy.

SDCT is a novel imaging technology that, unlike conventional CT, is equipped with a dual-layer detector that allows for separate registration of low-energy and high-energy photons. The projection data simultaneously obtained from both detector layers can also be utilized to generate various diagnostically useful spectral images, including, virtual monoenergetic images (display tissue attenuation properties similar to those resulting from imaging with a mono-energetic beam at a single energy level and virtual unenhanced images (tissue decomposition is used to remove iodine content from an image to produce a no iodine image). The exponential dependence on the atomic number (Z4) and proximity of iodine’s K-edge (approximately 33 keV) results in increased contrast in low-energy VMIs (VMIlow) on contrast-enhanced CT scans [2-4].

The coining of the term “tumoral calcinosis” (TC) dates back to 1943 and is attributed to Inclan et al., who reported three cases of TC that involved the gluteal area, the elbows, and in one case the thigh and hip. All TC reports were described in patients of Black and Hispanic ethnicities who were under 20 years of age, and, in two cases out of three, women were affected. The histopathological examination of the tumors revealed a multicystic structure and calcium salts deposits. Treatment consisted of surgical resection of the masses with a favorable evolution of the patients [1]. Giard and Duret also reported this disease in previous French medical literature [5,6]. In German journals, TC was known as Teutschlaender’s disease [7,8].

TC can be either primary or secondary. Primary TC can be further classified into two subtypes: hyperphosphatemic TC and normophosphatemic TC. Primary hyperphosphatemic TC is familial, and mutations in the Fibroblast Growth Factor 23 (FGF23), GalNAc Transferase 3 (GALNT3), or KLOTHO genes are critical factors in its genetic basis, resulting in reduced phosphorus elimination [9]. Primary normophosphatemic TC is also believed to be familial and is caused by deficiencies in the SAMD9 protein. Local traumatic lesions and the intake of phosphorus-based laxatives are thought to play a role since both lead to a state of transient hyperphosphatemia [2,8]. Calcium levels are normal in both subtypes, and phosphate levels are elevated in the hyperphosphatemic subtype [8,10]. Vitamin D levels are elevated, and PTH response is typical in these patients [11,12].

Secondary TC develops mainly in patients with ESRD. Such subjects often develop concurrent secondary or tertiary hyperparathyroidism [5]. ESRD/hemodialysis-related TC is characterized by hyperphosphatemia and in 75% of cases by hypercalcemia [10,11]. Uremic patients can also suffer from calcinosis circumscripta (CC), which differs from TC with regard to localization (CC: subcutaneous tissue, TC: bursae) and evolution (TC is more extensive than CC) [12]. Our patient showed lab features of secondary TC, as evidenced by tertiary hyperparathyroidism at 2,300.2 pg/mL (reference range: 18.5-88 pg/mL), phosphorus at 7.8 mg/mL (reference range: 2.5-4.9 mg/mL), calcium at 8.9 mg/mL (reference range: 8.6-10.6 mg/mL) and calcium-phosphorus product at 69.42 mg^2^/dL^2^. Secondary TC is a rare event in ESRD [13] and, in our report, leads to significant depositions of hydroxyapatite in the manubrium and suprasternal space of a patient with uremia and prior surgical trauma. Previous reports of TC in the sternoclavicular joints have also been described [14].

Diagnosis of both primary and secondary TC can be made by combining the clinical scenario and typical radiological features with the serum biochemical profile. Patients present with painless periarticular masses that cause no general symptoms and involve mainly the hips, elbows, or shoulders. Plain radiographs reveal the presence of well-defined nodules with fibrous septa (chicken-wire or cobblestoned appearance). Calcium layering results in the “sedimentation sign,” which can be highlighted using a horizontal beam radiological examination, computed tomography, and even magnetic resonance imaging. If bone scintigraphy is employed, TC sites exhibit high uptake. Ultrasound examination is useful in detecting fluid collections [8,10,12]. In our case, the diagnosis was made based on a CT scan and a metabolic profile. A full body multispectral nuclear fusion scan was done later to detect other masses.

Treatment strategies differ depending on the TC subtype. Surgical resection of the tumor is the procedure of choice in primary TC, yet recurrence has been noted in many cases. In patients with elevated levels of serum phosphorus, other measures might be useful: diets with low intake of phosphorus, acetazolamide to increase urinary phosphate elimination, and oral phosphate binders such as aluminum hydroxide or sevelamer carbonate. Combined surgical and medical treatment seems to generate the best results [8,10,12].

In ESRD-related TC, treatment is focused on the pathophysiological mechanisms driving this subtype: serum calcium-phosphorus product above 45 mg^2^/dL^2^, which leads to calcium salts deposits in soft tissues. Thus, medical treatment based on diets deficient in phosphorus, dialysis, and phosphate binders (but not based on aluminum salts) are used. When unsuccessful, it is replaced by surgery. The surgical approach consists of subtotal or total parathyroidectomy and tackles the encountered masses only in peculiar clinical settings. ESRD patients also benefit from renal transplantation [10,12].

## Conclusions

The diagnosis of TC can be made by combining the clinical scenario and typical radiological features with the serum biochemical profile. Spectral CT reconstruction can serve as a problem-solving tool, helping in better diagnostic confidence, and may reveal additional significant findings, such as enlarged ectopic parathyroid gland, as in our patient. Additionally, there is no extra radiation dose or specific imaging protocols needed for SDCT.
